# Understanding the Immune System and Biospecimen-Based Response in Glioblastoma: A Practical Guide to Utilizing Signal Redundancy for Biomarker and Immune Signature Discovery

**DOI:** 10.3390/curroncol32010016

**Published:** 2024-12-28

**Authors:** Luke R. Jackson, Anna Erickson, Kevin Camphausen, Andra V. Krauze

**Affiliations:** Radiation Oncology Branch, Center for Cancer Research, National Cancer Institute, National Institute of Health, 9000 Rockville Pike, Building 10, CRC, Bethesda, MD 20892, USA; luke.jackson@nih.gov (L.R.J.); anna.erickson@nih.gov (A.E.); camphauk@mail.nih.gov (K.C.)

**Keywords:** immune system, immune response, glioblastoma, biomarkers, tumor resistance

## Abstract

Glioblastoma (GBM) is a primary central nervous system malignancy with a median survival of 15–20 months. The presence of both intra- and intertumoral heterogeneity limits understanding of biological mechanisms leading to tumor resistance, including immune escape. An attractive field of research to examine treatment resistance are immune signatures composed of cluster of differentiation (CD) markers and cytokines. CD markers are surface markers expressed on various cells throughout the body, often associated with immune cells. Cytokines are the effector molecules of the immune system. Together, CD markers and cytokines can serve as useful biomarkers to reflect immune status in patients with GBM. However, there are gaps in the understanding of the intricate interactions between GBM and the peripheral immune system and how these interactions change with standard and immune-modulating treatments. The key to understanding the true nature of these interactions is through multi-omic analysis of tumor progression and treatment response. This review aims to identify potential non-invasive blood-based biomarkers that can contribute to an immune signature through multi-omic approaches, leading to a better understanding of immune involvement in GBM.

## 1. Introduction

Glioblastoma (GBM) is a primary malignancy of the central nervous system with a median survival of 15–20 months with current standard of care, including chemotherapy, external beam radiation, and maximally safe surgical resection [1]. Tumor-treating fields have recently been found to offer a survival benefit of approximately three months, but despite all currently available treatment options, recurrence is nearly universal, with a 17% survival rate at two years and 10% at five years [2]. Central to the difficulty in treating GBM is the presence of molecular heterogeneity both within individual tumors and between tumors from different patients [3], limiting understanding of biological mechanisms that lead to tumor resistance, including immune escape. To improve treatment response through tumor-specific management, molecular profiling of the diverse cell populations within the tumor has been explored and is critical to advancing outcomes for patients with GBM [4].

Cancers have distinct alterations in their cellular physiology relative to non-neoplastic tissue, including immune signatures linked to tumor resistance [5]. These alterations in cellular function introduce the possibility of detecting changes in DNA, RNA, proteins, or other biomolecules which serve as biomarkers that provide information to inform diagnosis, management, and prognosis [6]. Biomarkers can be detected from tumor tissue directly, or measured following release of cellular products into the bloodstream, enabling liquid biopsies as alternatives to tissue biopsy and imaging as modalities to monitor GBM treatment response and therapy optimization [7,8]. High sensitivity and specificity liquid biopsy biomarkers can lead to an earlier diagnosis, prognosis, and more tailored therapies [9]. An additional benefit of cerebrospinal fluid (CSF) or blood-based biomarker detection is longitudinal non-invasive monitoring of GBM for recurrence and progression; however, no formal approval for liquid biopsies in GBM diagnosis and therapy monitoring by regulatory agencies currently exists [10]. Several biomarkers are used to classify GBM, including genetic alterations [11] and transcriptional/RNA profiling [12], and studies have linked these biomarkers with differences in treatment responsiveness [13]. An attractive field of research to examine immune signatures for treatment resistance are cluster of differentiation (CD) markers and cytokines. CD markers are surface markers expressed on various cells throughout the body, characteristically immune cells, and play a role in cell signaling crosstalk and induction [14]. CD markers can be employed for treatment monitoring as well as therapy in some cancers but have remained investigational in GBM. Cytokines are also of interest in GBM since tumor cells have been found to produce them, ultimately leading to systemic immunosuppression in patients [15,16]. Despite documented associations between CD markers/cytokines and treatment resistance in GBM, with connections to immunosuppression and evasion leading to therapy resistance, there is currently no biomarker in GBM to capture immune response or relate to clinical management, including radiation therapy (RT) or chemotherapy with temozolomide (TMZ). Since tumors can be difficult to access for biopsy or resection and tissue biopsies are limited by sampling bias with ensuing inaccurate capture of tumor heterogeneity, leveraging serum immune signatures may prove pivotal in GBM. This review is aimed at (1) defining immune subsets in the brain and glioma, (2) characterizing current data surrounding immune signatures in GBM, (3) describing linkages between omic and immune signatures, and (4) discussing potential therapeutic implications and eventual implementation into clinical practice.

## 2. Immune Subsets in the Brain and Glioma—Summarizing the Role of the Immune System in GBM

**Figure 1:**
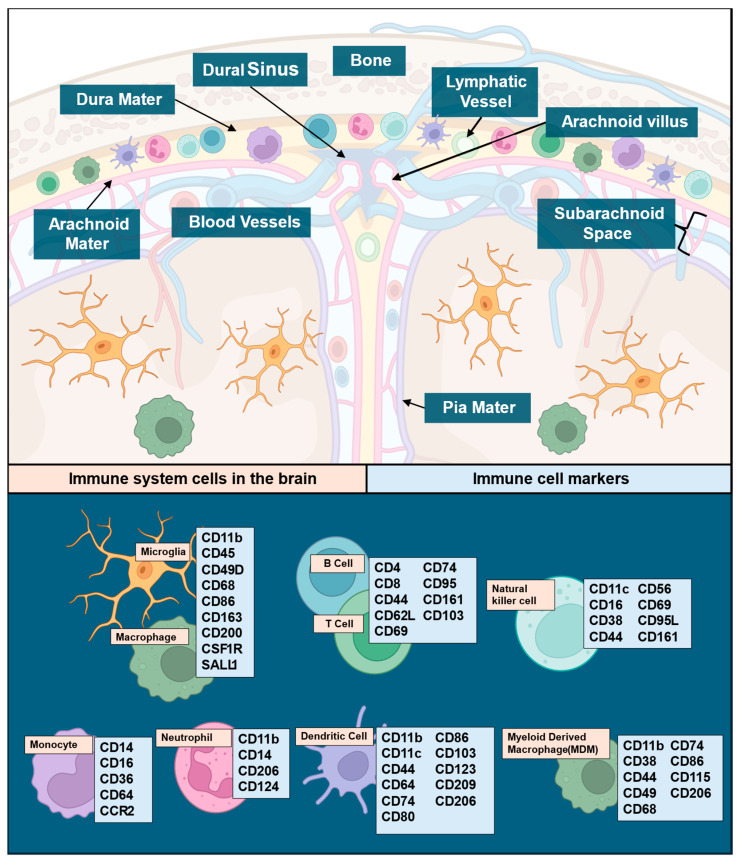
The brain as a site of active and passive immunity illustrating known linkages to cell types and their immune functions. Immune cells in the brain and their location (**top panel**), legend of immune cell types, and their respective markers (**lower panel**). While other immune cells, like mast and plasma cells, are known to play a role in CNS immune surveillance, there is limited data available demonstrating involvement in glioma, and their cell makers are redundant with the other cells listed here. Immune maker signatures were based on data established in previously published reviews [25,26,27,28,29,30,31]. Illustrations in this figure were created with Biorender.com (accessed on 23 September 2024).

The central nervous system (CNS) is generally considered immune privileged, which is essential given its limited regenerative capacity. However, this ‘privilege’ is not a complete absence of immunity but rather an intricate regulation of modified active and passive immunity [17]. The CNS is under close immune surveillance for abnormal cellular processes and pathogens by monitoring immune cells that are resident in the surrounding meninges and CSF [17,18,19,20], including dendritic cells (DCs), border-associated macrophages, T cells, innate lymphoid cells, neutrophils, and B cells [21,22,23]. These sites serve as the primary method by which CNS-derived antigens are presented to the peripheral immune system [24], activating adaptive immune responses and allowing for CNS–immune system crosstalk (Figure 1).

**Figure 2:**
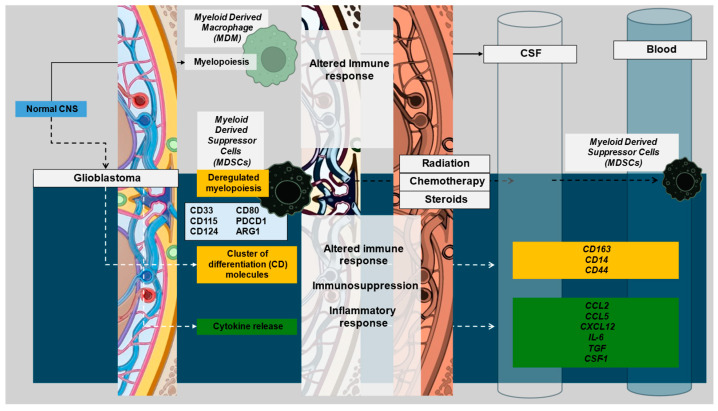
Glioblastoma development leads to ambivalent alteration in immune function with ensuing evolution of immune marker profiles as consequences of tumor progression, biological aggressiveness, and subsequent management. These are reflected in immune signatures of varying degrees in tissue, CSF, and blood. Illustrations in this figure were created with Biorender.com and PowerPoint.

Immune cells and their respective markers can represent an avenue for cell-specific immune signal attribution (Figure 1), particularly since basal CNS immune cell populations are subject to change upon pathological insults like GBM. The microenvironment in GBM is heterogeneous, consisting of microglial cells, neuronal cells, fibroblasts, pericytes, astrocytes, immune cells (microglia/macrophages, TILs, NK cells, neutrophils, MDSCs, and DCs), and soluble cytokines secreted by various cells. Prior reviews have comprehensively covered the distributions of immune cells and their functions within the CNS [25,26,27,28,29,30,31]. The scope of this review will remain focused on cell markers and cytokines as they relate to immune involvement in GBM. The main component of innate immunity within the CNS are microglia, which make up 5–12% of the CNS and serve as phagocytes within the brain [32]. Microglia and macrophages together form tumor-associated macrophages (TAMs) in GBM, constituting ~30% of tumor mass and the main tumoral immune cells [33]. Microglia release factors such as stress-inducible protein 1 (STI1), epidermal growth factor (EGF), or transforming growth factor beta (TGF-β), which increase glioma proliferation, migration, and invasion [34]. TAM accumulation in GBM has been found to correlate with tumor grade [35]. In the TME, TAMs secrete low levels of inflammatory cytokines and lack the ability to aid in T cell responses via co-stimulation, contributing to the immunosuppressive nature of GBM [36,37]. While T cells make up less than 0.25% of tumor cells, they are the primary lymphoid component of the tumor microenvironment (TME) [38], and T cell populations from both glioma specimens and peripheral blood mononuclear cells in primary and recurrent glioma exhibit variations in T cell subgroups. Myeloid cells also involved in GBM include natural killer (NK) cells, neutrophils, and dendritic cells (DCs). NK cells recognize and respond to abnormal cells via antibody-dependent cellular cytotoxicity or direct lysis by releasing cytokines in the TME [39]. NK cell activation is impaired when macrophages adopt a pro-tumoral phenotype, but no such link has been identified in GBM [40]. Most GBM patients have neutrophilia [41], leading to studies that have evaluated the neutrophil–lymphocyte ratio (NLR) as a biomarker. Higher-grade tumors, poor prognosis, and poorer overall outcomes are correlated with increased NLRs in glioma [42,43,44,45,46]. Interestingly, a high neutrophil count prior to treatment correlated with positive initial responses to bevacizumab [47]. However, increased infiltration of neutrophils in later stages of diseases promoted GBM transition to a mesenchymal phenotype favoring invasion and resistance to anti-VEGF and radiation therapies [48]. DCs, a group of antigen-presenting cells linking adaptive and innate immune responses [49], are present in the immunological niches surrounding the CNS and aid in activating the peripheral immune system under inflammatory conditions [50,51]. This aspect may facilitate the measurement of peripheral antigens in serum [52]. Intertumoral plasmacytoid DCs have been reported as a poor prognostic factor in some malignancies and were also found elevated in glioma patients, particularly if they presented with aphasia [53]. This raises the possibility that they may play a role in the pathogenesis of gliomas. While the specific role of DCs in glioma remains an area of active study, current research suggests that DCs are functionally connected to microglia, macrophages, T cells, and tumor cells within the microenvironment [54]. Neoplastic conditions can induce deregulated myelopoiesis, resulting in the production of immature myeloid-derived suppressor cells (MDSCs) that circulate in peripheral blood and land in the tumor bed [55,56,57] (Figure 2). In both immunocompetent and immunodeficient preclinical small animal models of glioma and humans with glioma, elevated MDSC levels have been seen in the peripheral blood [58]. MDSCs can even be used as a blood-based biomarker for glioma recurrence [59]. However, there are discrepancies regarding the mechanism of immunosuppression by MDSCs in both animal models and humans due to differences in outcomes between biological sexes [60,61,62]. This aspect merits close attention in analysis of sex as a biological variable (SABV) in glioma, particularly since glioma incidence in males is higher than in females and additional data supports superior outcomes in females compared to males. While the mechanism for either aspect is likely a multifactorial finding, it has been shown to have associations with adaptive immune response and requires further study [63]. Cytokines, as the effector molecules of the immune system, are involved in GBM, the most important being CCL2, CCL5, CXCL12, IL-6, TGF-β, and CSF-1 [64] with each serving as expression inducers and downstream effectors (Table 1). The key cytokines identified in the literature also have the potential to serve as blood-based immune signature markers in GBM based on existing publications documenting their measurement in serum [65].

**Table 1:** Immunosuppressive cytokines in GBM and their downstream effects.

Cytokine	Expression Inducers	Downstream Effectors	References
CCL2	INF-γ IL-1 IL-4 IL-6 TGF-β TNF-α	↑ CCR5 → ↑ ARG-1 ↑ CCR2+ TAMs ↑ CCR4+ Tregs	[64,66,67]
CCL5	Inherent to GBM	↑ ARG-1 ↑ IL-10 ↑ PI3K/AKT activation ↑ mTOR activation in GSCs	[64,68,69,70,71]
CXCL12	TMZ Hypoxia	↑ CD45+, CXCR4+, MMP9+ myeloid cells ↑ CXCR4+ M2 TAM	[64,72]
IL-6	Hypoxia Chemotherapy RT	↑ M2 phenotype ↑ PD-L1 ↑ B7-H4 ↑ ARG-1 ↑ CD163 ↑ CD206	[64,73,74,75]
TGF-β	Inherent to GBM	↓ NK infiltration ↓ CD8+ T cells ↓ CD107a ↓ INF-γ ↓ TNF-α	[64,76]
CSF-1	RT	↑ M2 phenotype ↑ angiogenesis ↑ Factor H ↑ Factor H-like protein ↑ C1 inactivator ↑ CD59 ↑ CD46 ↑ CD55	[64,77,78,79,80]

## 3. Characterizing Immune Signatures in GBM

**Figure 3:**
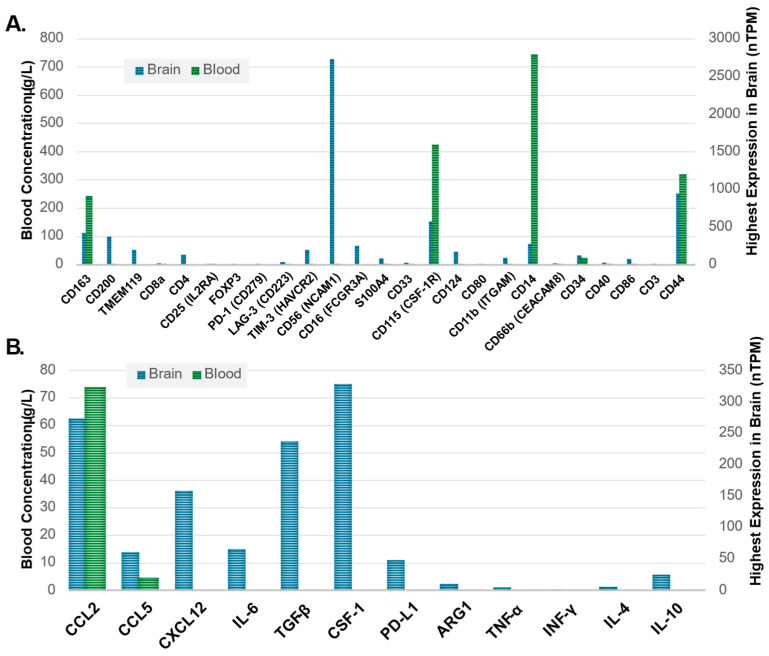
(**A**) Immune cell markers, clusters of differentiation (CD), and (**B**) cytokine markers illustrating expression in healthy brain tissue as compared to blood based on highest expression in the brain and concentration in the blood, respectively. Average blood concentration values (left Y-axis) were plotted next to the brain tissue-specific maximum transcript levels (right Y-axis) for each marker with data obtained from the Human Protein Atlas (HPA) [82] showing the differential expression of blood and tissues. The Human Protein Atlas data for both blood and brain tissue specimens are representative of patients without GBM to help identify which immune blood-based biomarkers may be able to reflect changes in brain tissue. The Human Protein Atlas. Available online: https://www.proteinatlas.org/ (accessed 23 September 2024).

Given the increasing importance of immune system activation and immunotherapy in treating systemic malignancies, it is imperative that immune signatures be characterized in GBM. Several options may be employed to accomplish this. Emphasis can arguably be placed on the use of noninvasive biospecimens such as blood, and several immune markers may represent attractive options for analysis given selective expression in the brain and potentially in brain tumors (Figure 2 and Figure 3).

There is observed differential or enhanced expression of several markers in blood relative to tissue representing promising candidates to watch in analyses of serum and plasma-based biospecimens (Figure 3A). CD14, CD44, CD115, and CD163 are well captured in the blood and brains of patients without GBM, providing a potential avenue for noninvasive biospecimen analysis and immune signature monitoring in GBM (Figure 3A). Cytokines with different levels of measurement in healthy brain tissue as compared to blood may also be useful as biomarkers in GBM (Figure 3B). CCL2 appears to hold the most potential due to its high expression in both the brain and blood at a basal state with evidence of alteration of the CCL2 and CCR2/MDSC axis in glioma and recent evidence supporting CCL2 and CCL7 inducing migration of MDSCs in the TME as well as association with a detriment in survival in GBM [81]. As seen in both Figure 1 and Figure 2, there are several overlapping immune cellular markers in GBM. However, each immune cell has differing functions in the context of GBM, highlighting the importance of understanding interaction complexities to derive meaning amongst the redundant signals. Additionally, differential expression between blood and brain with both cellular markers and cytokines (Figure 3) highlights specific candidate biomarkers for immune function in GBM. However, the findings in Figure 3 serve mainly as a method to identify which blood-based markers might be reflective of changes that occur in the brain. The data in Figure 3 is representative of patients without GBM and thus serves only as a means of hypothesis generation. For example, while CD14, CD115, and CD163 all show differential expression between the blood and brain in a non-diseased state (Figure 3A), their relative proportions are subject to change as the disease progresses. The same can be said for cytokines as well, with most having little to no expression in the blood in a disease-free state but have the potential to change significantly given the immunological changes that occur with GBM (Figure 3B). These combined features underscore the redundancy of immune system interactions in GBM and illustrate that untangling relationships between immune markers and downstream effects or linkages with molecular classification is possible provided the appropriate markers are employed given existing evidence and logical inference.

### 3.1. Characterizing Immunogenicity and the Immunologically “Cold Phenotype” in GBM

Several features alter the immunological state in patients with a GBM diagnosis, including patient-related factors, tumor presence/progression, and management with chemotherapy, radiation, and adjuvant corticosteroids to manage symptom control. Traditional chemotherapy and RT nonspecifically target rapidly dividing cells, including immune cells, further exacerbating tumor-related systemic immunosuppression [83] through direct or indirect mediators, utilizing cytokines as effectors and expression inducers (Figure 2, Table 1). The downstream effectors are highly complex, exhibiting wide-ranging signaling redundancy (e.g., PI3K/AKT activation, mTOR).

**Figure 4:**
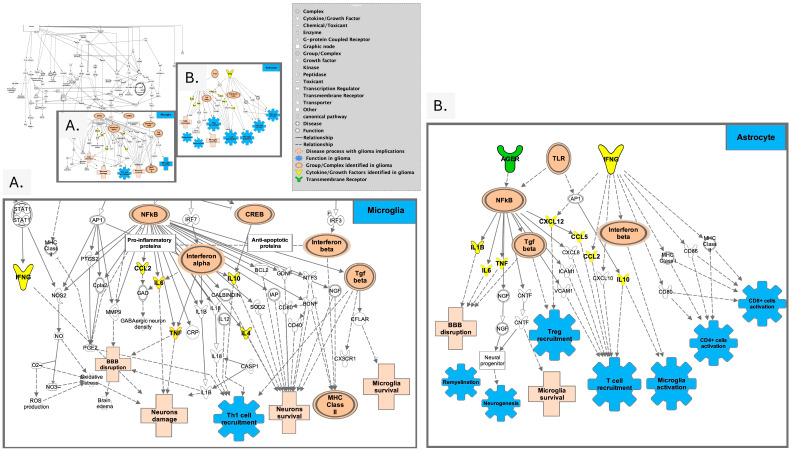
Overview of neuro-inflammation pathways. (**A**). Prominent signals associated with pro-inflammatory signaling (CCL2, IL6, TNF, IL10, and IL4) are illustrated downstream from NF-κβ in neuroinflammation pathways as identified in microglia. (**B**). Signals present in astrocytes in neuroinflammation leading to neurogenesis, Treg and T cell recruitment, microglial activation (adapted from Ingenuity Pathway Analysis (IPA)) [95].

Notably, peripheral blood analysis of patients undergoing chemoradiotherapy for glioma most commonly report lymphopenia during or after radio/chemotherapy with the best predictors for lymphopenia after chemotherapy initiation being low pre-therapy total lymphocyte count, advanced age, and female biological sex [84,85,86,87]. Radiation has a significant impact on immune cell survival, as lymphocytes are one of the most sensitive cells to radiation in the body, and standard radiation of 60 Gy delivered in 30 fractions for GBM causes toxicity in up to 98% of circulating lymphocytes [88]. Radiation, however, can also be immunogenic secondary to initiation of proinflammatory cytokine cascades, increased tumor-specific neoantigen production [89], and mutations that can induce changes in the T cell repertoire of TILs [90,91]. Interestingly, RT was found to induce type I interferon to increase the immunogenicity of an irradiated tumor, potentially serving as an additional biomarker [92,93,94], while TGF-mediated signaling may decrease interferon release (Table 1, Figure 4).

Dexamethasone and other steroids commonly employed for symptomatic management of GBM have been proven to exacerbate lymphopenia which may confound our current understanding of immune cell-based biomarkers [96,97,98]. Both in-vitro and ex-vivo studies have shown that steroids alter the maturation of DCs, resulting in hypo-responsiveness, further impacting T cell development, polarization, activation, and migration via impaired expression of cytokines, chemokines, and adhesion molecules, all promoting immunosuppression [99,100]. In pre-chemotherapy, pre-RT, and pre-resection patients with GBM, baseline lymphopenia is more frequently observed in patients who received dexamethasone for supportive treatment [101]. In addition, the primary immune cell populations, including CD4+ and CD8+ T cells, neutrophils, monocytes, and NK cells are significantly reduced in patients who receive dexamethasone, as are the total lymphocyte counts [102,103]. Steroid use in GBM has been linked to higher counts of blood MDSCs, which may further contribute to the immunologically cold phenotype of patients with GBM and represent an attractive feature for biomarker exploration.

It should be noted that several studies have looked at the other side of the immune relationship, exploring T-cell infiltration and patient outcome, with evidence for the degree of immune cell infiltration directly related to overall survival [104], including an eight-gene signature significantly associated with increased patient overall survival, which was validated and employed to predict clinical responses to anti-PD-L1 immunotherapy [44]. Characterization of the immune signature in GBM relating to M1 and M2 macrophages (Table 1) found that macrophage polarization towards the M1 state is associated with increased overall survival, while M2 polarization is associated with an impaired prognosis [105]. A study including recurrent glioma found that levels of NLR, red cell distribution width-to-platelet ratio in peripheral blood prior to re-resection, and abundance of macrophages in re-resected specimens were predictive of worse survival following surgery, with the macrophages exhibiting an increased expression of immunosuppressive genes [106]. Overall poorer survival outcomes are associated with the immunosuppressive and underactive immune phenotype displayed in both primary and recurrent GBM.

While existing data illustrates the potential limitations in interpreting findings related to immune system markers in patients with glioma, given interference from the administration of corticosteroids and the impact of treatment on immune signals throughout the natural history of the disease, it is important to note that there are avenues for study that allow for the interpretation of emerging biomarker data. Capturing steroid administration has been traditionally difficult to do robustly, and data normalization using steroid agnostic immune markers or housekeeping markers to normalize signal may also represent an option. An attractive avenue is to pursue markers that directly align with the immunologically “cold” phenotype and markers directly linked to GBM, evaluate these for their relationship to outcomes, and circumvent signal perturbation secondary to steroid interference.

### 3.2. Characterizing the Immune Signature for Pro-Neural-to-Mesenchymal Transition (PMT)

**Figure 5:**
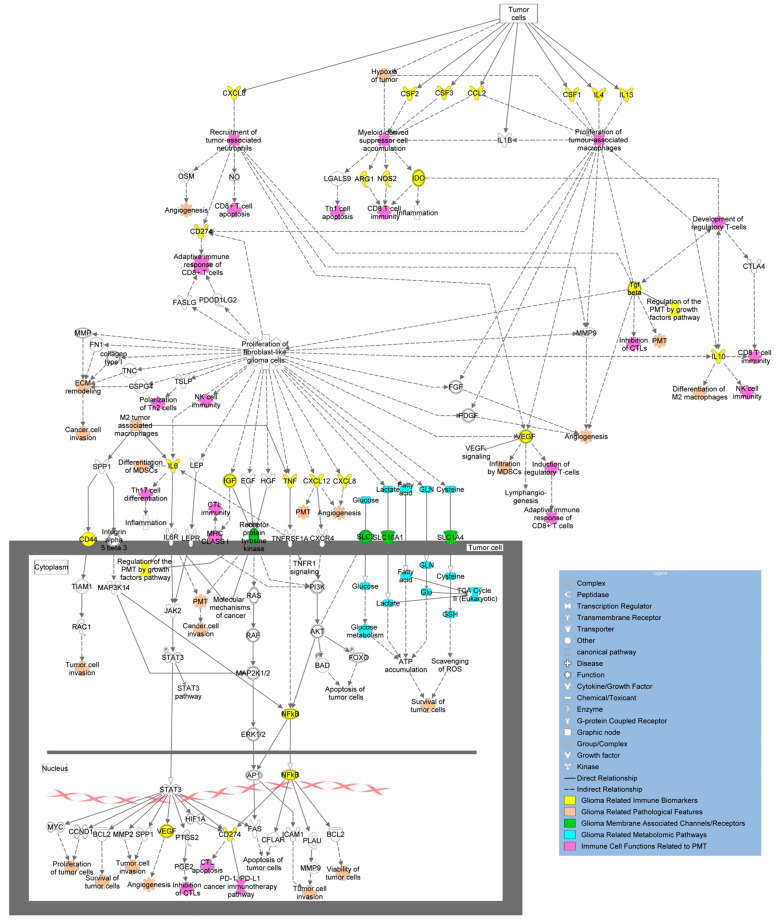
Overview of molecular pathways and immune signatures involved in PMT. Tumor cells produce cytokines, growth factors, and other relevant biomarkers (yellow) that increase invasive properties and have immunomodulating capacity, encouraging PMT. Effects of tumor immunomodulation (purple) and their impacts on tumor phenotypic behavior (peach) are linked above as well. Additional biomarkers are shown in the figure as transmembrane receptors and channels (green) and metabolites (blue) with their overall connections to immunomodulation and development of PMT (adapted from Ingenuity Pathway Analysis (IPA)) [95].

During treatment and disease progression, GBM undergoes changes to its molecular phenotype with pro-neural-to-mesenchymal transition (PMT), and the mesenchymal subtype is often associated with chemo- and radio-resistance, with a poorer prognosis as compared to the pro-neural type [107]. This phenomenon is attributed to the induction of signaling pathways and transcription factors found as extracellular stimuli in the TME [108] (Figure 5). Within the TME, TGF-β, tumor necrosis factor-alpha (TNF-α), interleukin 8 (IL-8), and Wnt all play an integral role in PMT [109]. This aspect can be exploited to create a signaling and immune signature for PMT. Data supports TNF-α as an important marker in its connection with GSCs, resulting in increases in the mesenchymal-specific marker CD109 [110]. Additional mesenchymal-associated markers that bear inclusion are CD44, CD97, ACTN1, EMP3, and CHI3L1 [109], and signaling molecules in the TME (ZEB1, TWIST1, FOXD1, and SNAI1) that have all been associated with PMT. Increased numbers of infiltrating lymphocytes were also found in the mesenchymal GMB, but the relative distribution of immune cell type is disputed [111,112]. TAMs are thought to be involved in PMT [41,113], with GBM signaling factors (cytokines, chemokines, soluble factors) activating and recruiting TAMs in the tumoral niche [114] where release of VEGF and CXCL2 promotes neovascularization [115]. GBM-derived CSF-1 also activates microglia, inducing angiogenesis via secretion of insulin-like growth factor binding protein 1 [116]. These GBM-specific secreted factors can lead vascular endothelial cells to adopt a mesenchymal phenotype, further promoting progression and chemoresistance [117]. Given that GBM remains adaptive and induces a highly immunosuppressive microenvironment through cytokines like NF-κβ, TGF-α, IL-8, and CXCL2, and signaling cascades like Wnt and others, these may serve as biomarkers of PMT and thus, markers of tumor resistance (Figure 5). However, this aspect also enforces the critical need to examine non-invasively acquired biospecimens that allow for monitoring of tumor behavior in real time versus the single time-point measurement of tissue-based approaches.

## 4. Linkages Between Omic and Immune Signatures and Novel Therapies

Overall, the systemic effects of cytokines and chemokines in GBM are highly complex, with critical evolving areas of study aimed at the linkages between the immune system, the metabolome, imaging findings, and outcomes. Analyses of metabolic products from the tumor and their effects are actively growing. The most widely used omics modality in GBM is transcriptomics, which has allowed for a more robust characterization of genetic heterogeneity in GBM [11,13,113] including emerging findings from multi-omic approaches where tumor–immune system interactions are being increasingly reported [118]. Cytokines are often identified and have been studied as potential biomarkers, revealing differences in GSC phenotypes, the ability to differentiate GBM and non-GBM patients, and alterations in CXCL ligands and acute-phase inflammatory markers after chemo-irradiation [65,119,120,121]. A comprehensive cytokine analysis involving large-scale proteomic data from GBM patients undergoing chemo-irradiation has yet to be reported but holds the potential to enhance the understanding of their measurement and downstream signaling cascade connections, as well as serve as an interesting comparison to existing data. In addition to proteins, studies in cancer biology have begun to look at the effects of metabolic products on immune cell function [122,123,124,125,126]. Amino acids and their metabolites play a critical role in immune cell function, altering proportions of T-cell subpopulations, B cells, and NK cells via modulation of the mTOR pathway. Macrophage phenotype (M1 vs. M2) is affected by amino acids and their metabolites, and studies with DCs and MDSCs show that arginine plays a significant role in immunomodulation [127]. Lipids, nucleic acids, and carbohydrates, each with their respective metabolites, have been involved in the modulation of immune cell function and its impact on the tumor microenvironment [123,125,126]. One recent study found that transcriptomic and proteomic analysis of mesenchymal-like GBM cells showed significantly elevated immune system activation with peroxisomal protein import and glycolysis, differing substantially from other GBM subtypes [128]. Another study performed an integrative proteo-lipidomic analysis resulting in the upregulation of proteins ASAH1, GPNMB, and SYNM in recurrent GBM, decreased neutrophil degranulation, IL-4 and IL-13 signaling, and immunoregulatory interactions involving lymphoid and non-lymphoid cells [129]. This study also identified the downregulation of ceramides and sphingolipids in recurrent GBM, which has been studied as a therapeutic target since ceramide processing relates to apoptosis resistance in GBM. Combining genomic and metabolomic information of primary GBM samples identified significant alterations in metabolites like lactic acid, glycine, and creatinine, alongside genetic alterations to stratify glioma into hypoxic, cell-cycle-specific, and immunomodulatory subclasses [130]. Proteogenomic analysis has further characterized GSCs, revealing low agreement between mRNA and protein expression levels in established gene sets used to classify GBM subtypes while providing further supporting evidence for novel proteomic signatures along the PMT axis that were associated with aggressiveness and poorer overall survival [131].

Tumor tissue-based immune signatures can represent highly conserved characteristics of malignancy and potentially represent an effective means of monitoring malignancy by defining immunogenicity in various malignancy settings [132]. GBM’s lack of representation in publicly available data has led to less than 5% of tissue available for analysis [133]. The TCGA data was recently employed to arrive at a nine-gene signature as a prognostic predictor in GBM [134]. An additional study has expanded upon this data and integrated multi-omic analysis to identify three distinct patterns of immune cell infiltration in GBM and its effects on treatment response and other clinical outcomes, but this study involved data that was obtained directly from tumor tissue samples, limiting comparison with serum or plasma. There is ongoing work in noninvasive immune function classification in glioma patients using high-throughput radiological imaging analysis via artificial intelligence (termed radiomics). Radiomics has been used to identify links between MRI tumor volume in glioma and clinical outcomes over time [135] and links to immune signatures/pathways [136]. A more comprehensive assessment of radioimmunomics and its use in GBM has been published [137]. These studies highlight the necessity for noninvasive multi-omic approaches to robustly and longitudinally characterize glioma phenotypes and immune system mechanistic connections.

## 5. Potential Therapeutic Implications and Eventual Implementation into Clinical Practice

While several advances in immunotherapy have succeeded in extracranial cancers, they have largely failed to translate to GBM due to a variety of reasons, including highly immunosuppressive TME, the presence of few TILs, most displaying an exhausted phenotype as well as poorly understood heterogeneity and plasticity at single-cell levels [12]. Additional barriers are low permeability across the BBB, TME evolution during treatment, and deficiency of immunogenic tumor antigens. Because immunotherapy in GBM has been investigated in many forms, including immune checkpoint blockers, therapeutic vaccines, oncolytic viral therapy, and chimeric antigen receptor (CAR) T-cell therapy [138,139], a rational approach to understanding immune signatures and immune biomarkers in GBM is essential to ensure advancement in preclinical studies that are investigating routes to improve immune response to GBM and clinical studies, including combination of standard-of-care therapies with immunomodulating agents, immune checkpoint inhibitors, or the use of oncolytic viruses [140,141].

**Figure 6:**
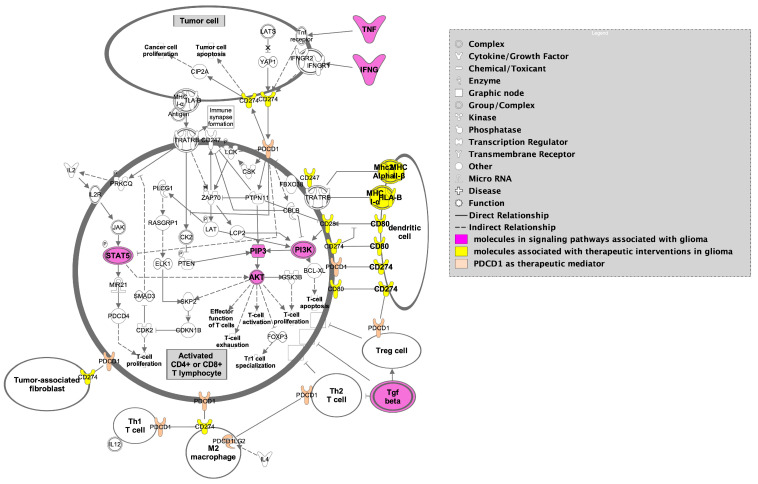
PD-1 immunotherapy illustrating dendritic cell markers (yellow) and the interplay between M2 macrophages, T cells, and tumor cells highlighting significant signaling pathways in GBM (magenta) in tumor cells and activated CD4+ and CD8+ T lymphocytes. Figure adapted from Ingenuity Pathway Analysis (IPA) [95].

An additional active area of study is the intersection of RT and immunogenicity, given that RT is one of the key facets of standard therapy for glioma. RT plays a significant role in immune activation, as previously noted; however, the combination of RT and immunotherapy in glioma yields mixed results [138]. Radiation-induced lymphodepletion may alter immune-mediated tumor control due to direct damage to lymphocytes, and the final interplay of immune response is not well understood [142]. Preclinical studies involving radiation and anti-PD-1 immunotherapy showed a significantly increased survival in the combination (radiation + anti-PD-1), and additional studies have found similar effects preclinically, involving combination radiation and immunotherapy (Figure 6).

However, in a meta-analysis of nine randomized phase II/III clinical trials, immunotherapy, while safe to combine with chemo-radiotherapy, did not significantly increase overall or progression-free survival [143]. Since then, additional studies have shown similar results involving RT and immunomodulatory therapy [144,145].

**Figure 7:**
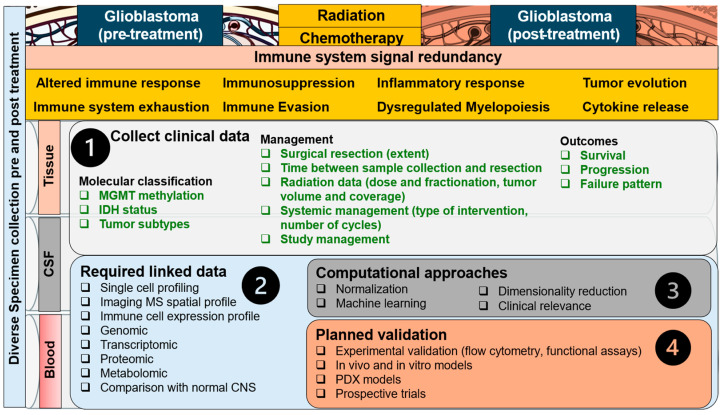
Approach to the immune signal redundancy problem in GBM. Signal redundancy is multifactorial and evolves in stages from the normal CNS to GBM development/progression/management (left to right), and with radiation and chemotherapy to encompass a balance of immune suppression, evasion, and response as well as immune system exhaustion (upper panel, left to right). The redundancy cannot be modified; thus, emphasis is placed on enhanced utilization of clinically available data (step 1), linkage of multi-channel data across all types of biospecimens (step 2), and comparison with the normal CNS. Computational approaches can then be employed to normalize data and select the most important clinically relevant features (step 3), followed by validation aimed at the most promising signals and the use of novel therapies (step 4).

Despite both RT and immunotherapy’s ability to garner an anti-tumoral immune response in extracranial cancers, they have largely failed to improve clinical outcomes in patients with GBM. Because of this, there is a critical need to investigate immune alteration in GBM to understand the current mechanisms at play for treatment resistance, recurrence, and ultimately, lack of translatable novel therapies in this otherwise lethal disease. Preliminary studies have identified CD markers and cytokines as regulators of pathogenesis and immunosuppression involved in GBM, but these are just pieces of a very complex, redundant, and sometimes conflicting signaling network (Figure 4, Figure 5 and Figure 6). Despite this, approaches exist that can clarify significance within signaling network interactions while considering appropriate controls, like multi-omic analysis with proteomic and metabolomic characterization due to their effects on cell–cell signaling and both tumor and immune cell phenotype. In these analyses, particular interest should be paid to makers of PMT (CD44, CD97, ACTN, EMP3, CHI3L1), M2 TAM/microglia (CD163, CD200, SALL1, CD115), and MDSCs (CD33, CD115, CD124, CD80, PD-L1, ARG1), in addition to cytokines associated with immunosuppression in GBM (NF-κβ, TGF-α/β, IL-8, CXCL2, CCL2, CCL5, IL-6, IL-4, IL-10, INF-α/I/γ, CSF-1, etc.) (Figure 3). Identifying a blood-based signature for PMT in GBM has the potential to identify malignant progression and treatment resistance, whereas traditional disease-tracking modalities like imaging lack specificity. Additionally, blood-based signatures to identify M2 TAM/microglia phenotype and MDSCs have the potential to reveal treatment susceptibility in patients with GBM and guide further research into disease-modifying therapies in this heterogeneous condition. Amino acids, lipids, and their respective metabolites are also of interest due to their ability to regulate immune cell function and alter the tumor microenvironment in the context of PMT and other pathological hallmarks of GBM. Longitudinal, non-invasive, multi-omic analyses of patient samples and associated clinical data have the potential to elucidate mechanisms behind tumor progression/treatment resistance in GBM, and through computational methods, provide context to clinically relevant features that will guide future experiments and clinical trials aimed at investigating GBM-induced immunosuppression (Figure 7). Multi-omic analytical workflows like those proposed here can also drive investigation and breakthroughs in extracranial cancers.

## 6. Conclusions

The Immune system’s role is highly conserved and can be exploited to both monitor disease and develop novel therapies with several promising molecules that are immune signature-defining in GBM. Biomarkers are currently lacking in GBM, with a pressing need to define immune signatures. Characterization of immune signatures will need to involve analyses aimed at patient outcomes along lines of exploration of the cold tumor phenotype, the PMT, and mechanistic connections with the proteome and metabolome to mitigate inherent complexity and redundancy related to patient factors, tumor burden, chemotherapy, radiation, corticosteroids, and evolving alteration in immune phenotype. Identifying biomarkers for specific interventions and understanding the immune signatures in GBM will allow us to better target treatments toward improving immune system-mediated clearance of tumors. The ability to do this effectively can provide transferable findings in other malignancies. Future research should be directed at biospecimen collection and targeted analysis of immune markers in GBM in conjunction with both standard care and management under clinical trials.

## Abbreviations

ARG1	arginase-1
ASAH1	N-acylsphingosine amidohydrolase 1
BBB	blood–brain barrier
CAR	chimeric antigen receptor
CCL2	CC motif ligand 2
CCL5	CC motif ligand 5
CCR4	C-C chemokine receptor 4
CCR5	C-C chemokine receptor 5
CD	cluster of differentiation
CNS	central nervous system
CSF	cerebrospinal fluid
CSF1R	colony-stimulating factor 1 receptor
CXCL12	C-X-C motif chemokine ligand 12
CXCR4	C-X-C motif chemokine receptor 4
DC	dendritic cell
DNA	deoxyribonucleic acid
EGF	epidermal growth factor
FOXD1	forkhead box D1
GBM	glioblastoma
GPNMB	glycoprotein nmb
GSC	glioma stem cell
Gy	gray
IL-1	interleukin 1
IL-4	interleukin 4
IL-6	interleukin 6
IL-10	interleukin 10
IL-13	interleukin 13
INF-γ	interferon gamma
INF-I	interferon type I
MDSC	myeloid-derived suppressor cells
MRI	magnetic resonance imaging
mAb	monoclonal antibody
mRNA	messenger RNA
NF-κβ	nuclear factor kappa beta
NK	natural killer
NLR	Neutrophil-to-lymphocyte ratio
PD-1	programmed cell death protein 1
PD-L1	programmed cell death protein ligand 1
PI3K	phosphoinositide 3-kinase
PMT	proneural-to-mesenchymal transition
RNA	ribonucleic acid
RT	radiation therapy
SALL1	Sal-like 2
SNAI1	snail family transcriptional repressor 1
STI1	stress-inducible protein 1
SYNM	synemin
TAM	tumor-associated macrophages
TCGA	The Cancer Genome Atlas
TGF-β	transforming growth factor β
TIL	tumor-infiltrating lymphocytes
TMEM119	transmembrane protein 119
TME	tumor microenvironment
TMZ	temozolomide
TNF-α	tumor necrosis factor α
TWIST1	twist family bHLH transcription factor 1
VEGF-A	vascular endothelial growth factor A
ZEB1	zinc finger E-box-binding homeobox 1
